# Evaluation of effectiveness of instruction and study habits in two consecutive clinical semesters of the medical curriculum munich (MeCuM) reveals the need for more time for self study and higher frequency of assessment

**DOI:** 10.1186/1472-6920-11-62

**Published:** 2011-08-26

**Authors:** Sophia Mueller, Nina Weichert, Veit Stoecklein, Ariane Hammitzsch, Giulia Pascuito, Christian Krug, Matthias Holzer, Mona Pfeiffer, Matthias Siebeck, Ralf Schmidmaier

**Affiliations:** 1 Institute of Clinical Radiology, University of Munich Hospitals, Marchioninistrasse 15, 81377 Munich, Germany; 2 Faculty of Medicine, Ludwig Maximilians University Munich, Bavariaring 19, 80336 Munich, Germany; 3 Department of Pediatric Oncology and Hematology, Charité Universitätsmedizin, Augustenburger Platz 1, 13353 Berlin, Germany; 4 Medizinische Klinik Innenstadt, Klinikum der Universität München, Ziemssenstrasse 1, 80336 Munich, Germany; 5 Chirurgische Klinik und Poliklinik Innenstadt, Klinikum der Universität München, Nussbaumstrasse 20, 80336 Munich, Germany

## Abstract

**Background:**

Seven years after implementing a new curriculum an evaluation was performed to explore possibilities for improvements.

Purposes: To analyze students' study habits in relation to exam frequency and to evaluate effectiveness of instruction.

**Methods:**

Time spent on self study (TSS) and the quantity of instruction (QI) was assessed during the internal medicine and the surgical semester. Students and faculty members were asked about study habits and their evaluation of the current curriculum.

**Results:**

The TSS/QI ratio as a measure of effectiveness of instruction ranges mainly below 1.0 and rises only prior to exams. Students and teachers prefer to have multiple smaller exams over the course of the semester. Furthermore, students wish to have more time for self-guided study.

**Conclusions:**

The TSS/QI ratio is predominantly below the aspired value of 1.0. Furthermore, the TSS/QI ratio is positively related to test frequency. We therefore propose a reduction of compulsory lessons and an increase in test frequency.

## Background

Following the new German "Regulation of the Licensing of Doctors" (Approbationsordnung für Ärzte) conducted by the German Federal Ministry of Health in 2002 [1], most German medical schools had to initiate profound modifications of their curricula. In order to fulfill the new requirements, Ludwig-Maximilians University (LMU) Munich implemented a new Medical Curriculum in Munich (MeCuM) in 2003 [2]. In general, medical studies continue to be divided into a preclinical coursework focusing on basic science, and a clinical portion of four years. The clinical portion of MeCuM has been split into 6 modules that are primarily subject-based. Module 2 covers internal medicine and Module 3 is dedicated to operative medicine. The presented analysis focuses on these two basic clinical specialties. Internal surveys repeatedly showed that students and teachers are generally satisfied with the internal medicine and the surgical semester. Nevertheless, we hypothesize that the current curricular structure and mode of assessment, which entail many mandatory courses and a low frequency of testing, do not motivate students to study continuously throughout the academic year.

In order to verify this hypothesis with regard to planned curricular changes, we conducted systematic surveys on students' study habits in internal medicine and surgical courses. We furthermore collected data enabling us to calculate the mean ratio of time for self study (TSS) and quantity of instruction (QI) as a tool to evaluate effectiveness of instruction [3]. For example a TSS/QI of three means, that for every hour of instruction, students spend three hours on self-study. TSS/QI has been shown to positively correlate with achievement and can therefore be considered a parameter for effective instruction [3]. Our assumption that students don't necessarily learn more with more mandatory courses has previously been proposed by others [4,5] and was recently strongly supported by the results of a study by Schmidt et al. [6]. Analyzing a total of 14000 medical students enrolling in 8 Dutch medical schools between 1989 and 1998 this study could show that time available for self-study was the only determinant of academic achievement as assessed by graduation rate and study duration. Lectures by contrast were negatively related to self-study time and graduation rate and positively related to study duration [6].

## Methods

### Survey 1 and 2

All students of four consecutive surgical semesters and all students of four consecutive internal medicine semesters were provided with an anonymous online questionnaire every week of the semester. Each semester consists of 15 weeks. Students were asked to give the amount of weekly mandatory lessons and the amount of time spent on self-study.

For each week of the surgical and the internal medicine semesters, the mean ratio of time spent on self-study (TSS) and quantity of instruction (QI) [3] was calculated.

A Wilcoxon rank sum test with continuity correction was used to compare TSS/QI in the group with 1 assessment per semester and the group with 2 assessments per semester. All valid measurements were used for a global test, and multiple comparisons were carried out at semester weeks 1, 8 and 15. Bonferroni correction as an adjustment for multiple testing was applied.

### Survey 3

Questionnaires were sent to students of the LMU Medical School who had recently finished the internal medicine and the surgical semester. The students received a link to an online version of the questionnaire, enabling them to complete the form anonymously for a two-week time period.

Additionally, questionnaires were collected from faculty members of the Departments of Internal Medicine at LMU Munich and teachers of all specialties involved in surgical courses. Internal medicine teachers were provided with a link to an online version of the questionnaire, which they could complete anonymously for a two-week time period. Staff members of the surgical classes were invited to complete the survey anonymously in written form during their morning report. The design of the online surveys ensured that the participant could only proceed to the next question after having answered the previous item.

The student questionnaire (SQ) contained 37 items. Possible answers for these questions were given on a six point Likert scale, ranging from "I strongly agree" to "I strongly disagree". The faculty questionnaire (FQ) consisted of 36 items. Answers for these questions were given on a four point Likert scale, ranging from "I strongly agree" to "I strongly disagree". We report the results of the items most relevant to the evaluation of students' study habits and the overall satisfaction within the internal medicine and the surgical semester.

### Statistical analysis

Statistical analysis was performed using the Statistical Package for the Social Sciences (SPSS 17.0, SPSS Inc., Chicago, IL). Figures were created using R 2.9.0 (The R Foundation for Statistical Computing, Vienna, Austria).

Descriptive presentation of data is shown as the distribution of answers in percent, and the means derived from all answers to each question. To subsume students' perceptions, the participants' responses were partially classified into two categories, "agreement" (including "I strongly agree", "I agree", "I rather agree") and "disagreement" (including "I rather disagree", "I disagree", "I strongly disagree"). Accordingly, teachers' responses were classified into "agreement" (including "I strongly agree", "I rather agree") and "disagreement" (including "I rather disagree", "I strongly disagree").

## Results

### Survey 1

In the four surgical semesters all students (n_1 _= 200, n_2 _= 226, n_3 _= 255, n_4 _= 212) were provided with a questionnaire every week of the semester resulting in 13,395 distributed questionnaires. The return rate was 28.8% (n = 3785). 1,976 complete and plausible pairs of TSS and QI were obtained, which equals 14.8% of all distributed questionnaires in the surgical semester.

**Figure 1:**
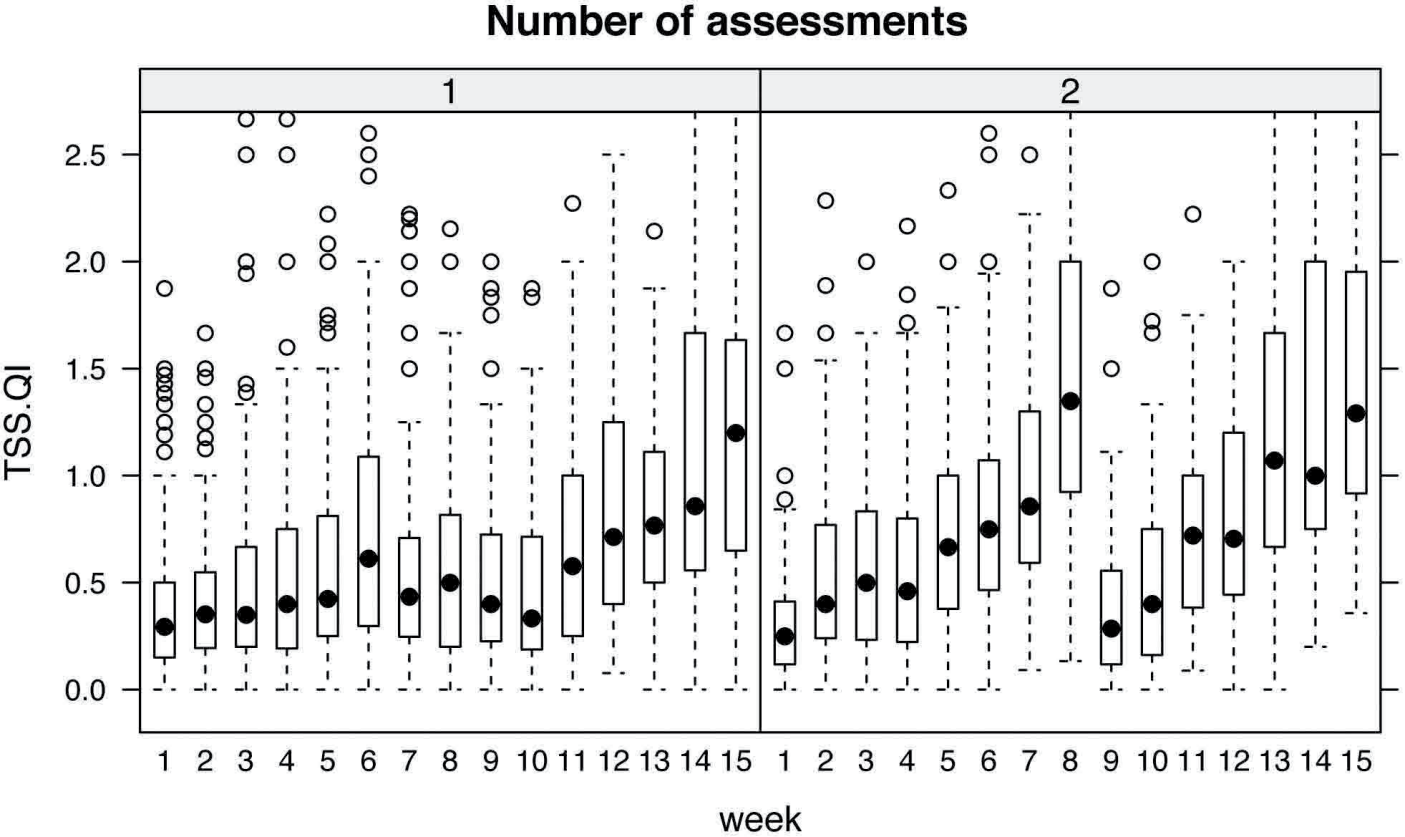
**Time of self-study divided by quantity of instruction (TSS/QI) over time (semester weeks)**. Left panel: Box-and-whisker plots, 1 assessment per semester (1976 data points, surgical semester), right panel: 2 assessments per semester (1284 data points, internal medicine semester). Median TSS/QI (black dots) is below 1 during the semester and increases above 1 only before assessments. Groups were significantly different when compared globally and at 8 weeks, and not different when compared at 1 week and at 15 weeks (adjustment for multiple testing was applied).

The mean TSS/QI ratio during the surgical semester mainly ranged clearly below 1.0, most of the weeks even below 0.5. In weeks 12 till 14 TSS/QI was increased. Only in week 15 did the TSS/QI exceed 1.0 (Figure 1).

### Survey 2

In the four consecutive internal medicine semesters, all students (n_1 _= 234, n_2 _= 230, n_3 _= 266, n_4 _= 232) were provided with a questionnaire every week of the semester, adding up to a total of 13,930 questionnaires. The return rate was 10.8% (n = 1,499). 1,277 complete and plausible pairs of TSS and QI were obtained, which equals 9.2% of all distributed questionnaires in the internal medicine semester.

The mean TSS/QI ratio during the internal medicine semester was predominately below 1.0 for most of the weeks. The only weeks where TSS/QI exceeded 1.0 were week 8 and weeks 13-15. In these weeks, the TSS/QI ratio ranged between 1.0 und 1.5 (Figure 1).

### TSS/QI comparison between groups

**Figure 2:**
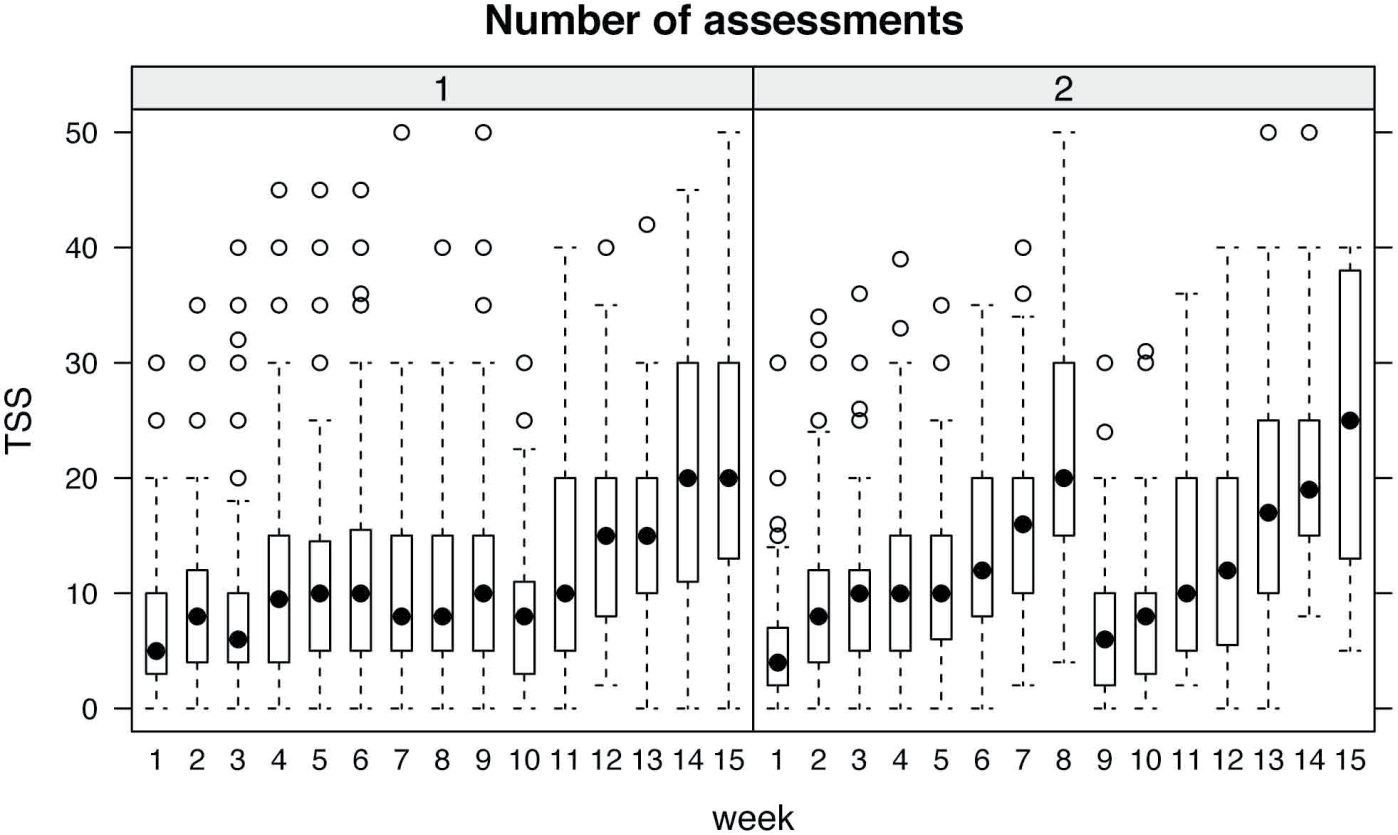
**Time of self-study over time (semester weeks)**. This figure illustrates that TSS/QI is mainly dominated by TSS in the internal medicine semester as well as in the surgical semester. Left panel: 1 assessment per semester (1976 data points, surgical semester), right panel: 2 assessments per semester (1284 data points, internal medicine semester).

**Figure 3:**
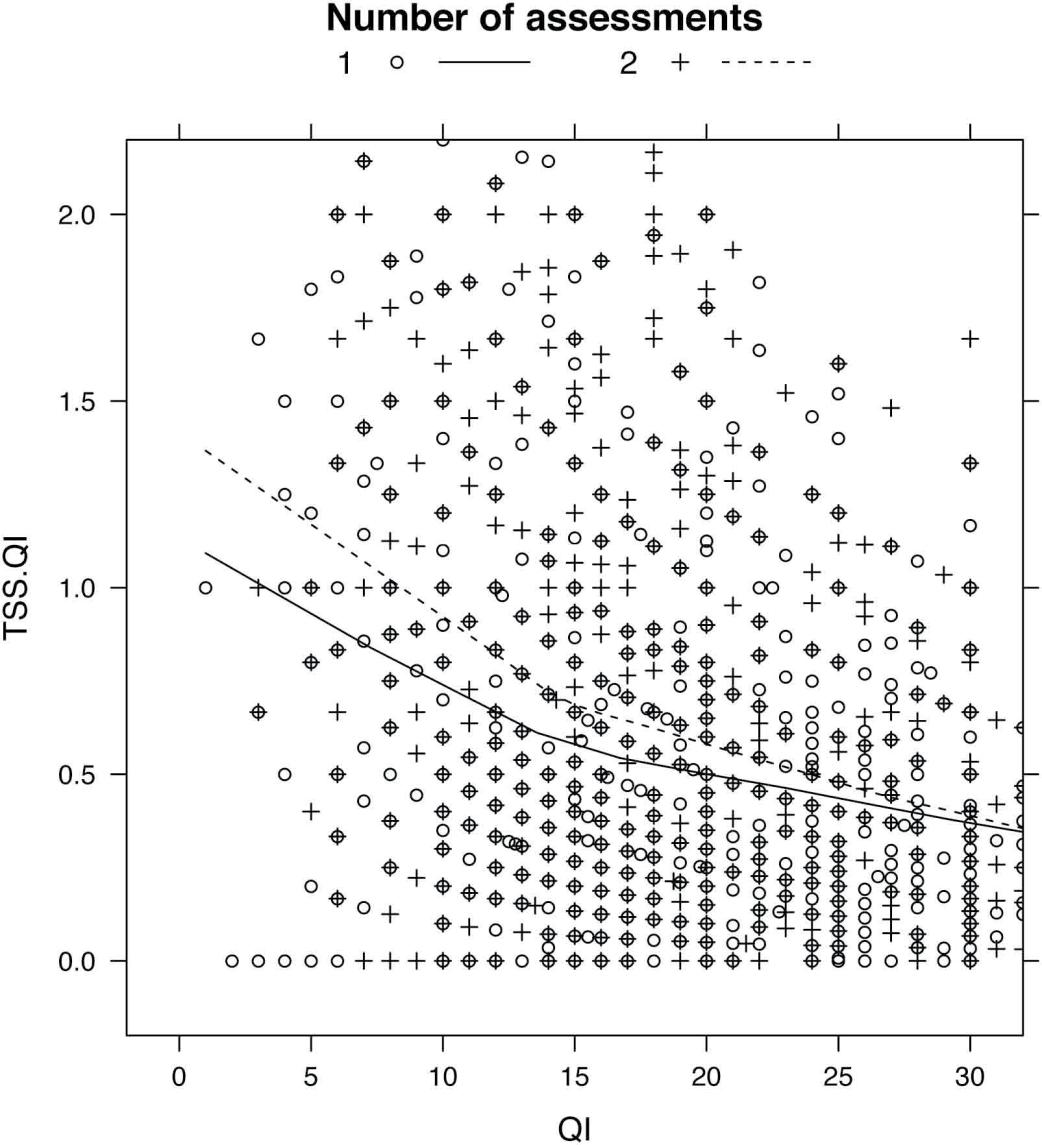
**Time of self-study divided by quantity of instruction (TSS/QI) over quantity of instruction (QI)**. 3,260 data points were smoothed using the LOESS algorithm. Open circles. and the solid line denote values from participants in the semester with one (1) assessment, plus signs and the dashed line are from participants with two (2) assessments per semester. TSS/QI and QI appear as inversely related, and TSS/QI tends to be higher in participants with 2 assessments than in those with 1 assessment, given the same QI.

In both surveys TSS was strongly coupled to TSS/QI raise, indicating that these raises mainly result from an increase in time spent on self-study, rather than from a decrease in QI (Figure 2). TSS/QI differed between the group with one assessment and the group with two assessments per semester. Groups were significantly different when compared globally and at week 8, and not different when compared at week 1 and week 15. Adjustment for multiple testing was applied. For a direct comparison of TSS/QI ratio in the semester with one assessment and the semester with two assessments see Figure 3. TSS/QI and QI appeared as inversely related, and TSS/QI tended to be higher in participants with 2 assessments than in those with 1 assessment, given the same QI.

### Survey 3

**Figure 4:**
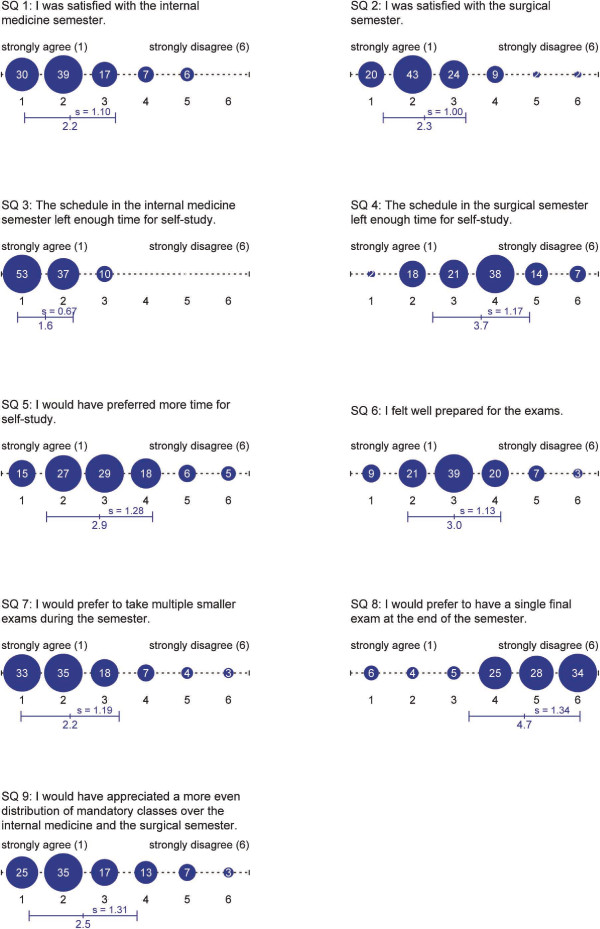
**Results of students' questionnaire**. Results are given in percent, mean and standard deviation, n (number of answers) = 109 for all questions.

**Figure 5:**
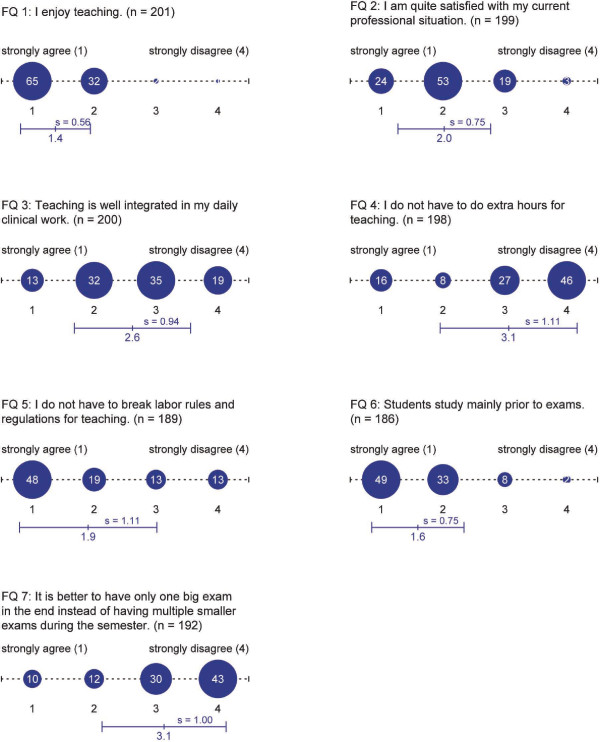
**Results of faculty members' questionnaire**. Results are shown in percent, mean and standard deviation, n (number of answers) is indicated for each question.

The response rate of the students' questionnaire was 26% (n = 109). The return rate of the questionnaires distributed amongst teachers in the internal medicine semester was 32% (n = 79). 122 data sets could be obtained from teachers in surgical specialities. A return rate could not be assessed. An overview of questions and results is shown in Figure 4 for students and Figure 5 for faculty members.

In general, most students were satisfied with the internal medicine and the surgical semester (Figure 4, SQ 1 and 2). Almost all faculty members enjoy teaching (Figure 5, FQ 1). The satisfaction with their current professional situation is quite high (FQ 2). They state that teaching is well integrated in their daily clinical work (FQ 3). Fulfillment of reaching obligations requires neither overtime nor infringement of labor rules and regulations (FQ 4 and 5).

Asking the students about their need of time for self-guided studies, most of them feel they have enough time in the internal medicine semester (SQ 3), whereas the majority thinks that they do not have enough time during the surgical semester (SQ 4). In fact, more than half of the students generally wish to have more time for self-guided studies (SQ 5). Additionally, students wish to have a more even distribution of compulsory lessons over the two semesters (SQ 9). Nevertheless, most students feel that the compulsory lessons provide good preparation for the exams (SQ 6).

Most students study mainly prior to exams, but many students would prefer to have multiple smaller exams during the semester (SQ 7) instead of one big final examination (SQ 8). Both statements are in line with the teachers' point of view (FQ 6). Teachers also state that it would be better to have multiple smaller exams during the semester instead of one final exam covering the entire semester's content (FQ 7).

## Discussion

The return rates of 26% for students and 30% for the internal medicine faculty members in survey 3, as well as a response rate of 28.8% in survey 1 can be considered as representative and are comparable to data on return rates for email surveys for university students at 21% as well as for surveys by regular mail at 32% [7,8]. The overall return rate in survey 2 of 10.8% seems quite low. However, results showed to be reproducible in four consecutive semesters. We therefore assume that these results are reliable.

The ratio of time spent on self study and quantity of instruction (TSS/QI) is an important measure for effective instruction as proposed by Gijselaers and Schmidt [3]. This concept dates back to the time-on-task hypothesis by Carol, which claims that it is time dedicated to studying that determines the learning outcome [9]. Given that medical curricula require processing a big quantity of information, it does not seem counterintuitive that it is crucial to provide students with enough time for self study that can be used for individual learning activities and memorization techniques. With regard to a future curriculum reform, TSS/QI would be a parameter that can be easily modified by reducing QI. Compared to other curricular measures seeking to improve learning outcomes and to increase effectiveness of instruction, like the reduction of group size [10,11] or a long-term mentorship between students and teachers [12], an adjustment of TSS/QI could be swiftly implemented and translated into improvement of the curriculum.

Our results show that TSS/QI is unacceptably low in our current curriculum (see Figure 1). This finding is particularly unfavorable when considering that, according to Gijselaers and Schmidt, TSS/QI is positively correlated to academic achievement. Additionally, Gijselaers and Schmidt found that students are not willing to spend more than 37 hours total per week on education. Given the low TSS/QI in our present curriculum, this suggests that our current instruction time is too high. Moreover, in our survey a majority of students wishes for more time for self-study and criticizes that under the present curriculum mandatory courses are not distributed evenly among the internal medicine and surgery semesters. Given the fact that some weeks of the present surgery semester leave almost no free time for self study this criticism appears justified. Therefore it can be concluded that reducing instruction time is necessary for future curriculum changes.

However, the relationship between mandatory lessons and TSS is not strictly linear, in other words we cannot simplify the relationship to the statement "the less we teach the more students study". Van der Drift et al. could show that the relationship is rather curvilinear, meaning that up to a certain extent scheduled lessons enhance TSS while mandatory courses beyond that point rather impede TSS [5]. In their study the ideal QI was found to lie between 8 and 10 hours per week, a value by far below what our current curriculum provides within most weeks. The German law exactly defines the minimum of mandatory instruction time required for sufficient guidance and study stimuli. These requirements are clearly below the quantity of instruction in our current curriculum. Accordingly it seems reasonable to redesign students' schedules so that QI does not to exceed German legal requirements and does not fluctuate significantly between and within semesters.

Apart from time available for self study, and necessary guidance to efficiently use this time, time actually spent on self-study also seems to be influenced by test frequency. Our data suggest that students study mainly prior to exams and that teachers and students consider multiple smaller exams during the semester preferable to one big exam in the end of the semester. The peak of TSS/QI ratio and TSS in week 8 of the internal medicine semester can be explained by a mid-semester exam. In the surgical semester, no such additional exam exists. A mid-semester peak of TSS/QI and TSS can therefore not be detected (Figures 1 and 2). In both semesters a clear increase of TSS/QI over 1 can be shown prior to the respective final exam. These results demonstrate the correlation between time spent on self-study and exam frequency. Findings in cognitive psychology studies in general and in medical education studies in particular further support our conclusion that test frequency should be increased when implementing a new curriculum. Testing is not merely able to serve as an assessment tool but has been shown to provide mnemonic benefits. These beneficial effects of testing on long term retention of information can mainly be attributed to two effects, a direct and an indirect one [13]. The direct effect results from students' awareness that they will be tested on the topics they are reading about or listening to in lectures. According to Larsen et al. this is an even stronger boost to later retention than repetition of the respective topic [13]. Additionally, the retrieval of knowledge itself strengthens long-term memory [14]. The indirect effects of testing result from increased study time and improvement of study strategies with regard to an up-coming exam. This phenomenon can be further promoted by more frequent testing (e.g. monthly) as students are prompted to adopt constant learning habits with an even distribution of study time over the academic year or semester. Studies demonstrate that evenly spaced study sessions promote memory performance [15]. Testing can serve as a tool to influence students learning habits likewise. Accordingly a meta-analysis of forty studies by Bangert-Drowns, Kulik and Kulik has shown, that frequent testing before a final exam results in better performance compared to less frequent (or no) testing prior to the final exam [16]. The effect on performance in exams which do not take place immediately after a course, but two to four years after a respective instruction unit (like the German medical licensing examination) might be more compelling and remains to be investigated. One further beneficial effect of exams is inherent in the process of test taking itself. Studies show that taking a test leads to better retention of information than restudying the material for an equivalent amount of time [17,18]. Apart from students' performance, there is evidence that more frequent testing improves students' evaluation of faculty [19,20].

Although our results are comprehensive and conclusions for further curricular changes can be drawn, there are several limitations to this study. One constraint is the relatively low response rate, especially in surveys 1 and 2 that might result in a selection bias, as highly motivated students could be over-represented. However, as those students are also likely to show a high willingness to dedicate time for self-study despite many compulsory lessons, this potential bias would presumably result in an overestimation of TSS/QI. As we claim that TSS/QI is too low in our current curriculum, this is biasing our results towards a more "conservative" estimate rather than towards incorrectly low TSS/QI values.

A second point of caution pertains to the outcome variables of our study. While we could show, that increased test frequency results in raised TSS/QI and TSS, that is increased quantity of learning, this study did not demonstrate the impact of TSS on learning outcome. Previous studies, even on a multicenter level [6] could already provide evidence for the positive relation between TSS and learning outcome [3,6]. However, future studies are recommended to correlate test frequency or TSS directly to exam results, levels of competence or graduation rates in order to verify that this evidence based assumption holds true.

It is also to note that this study exclusively focused on quantity of instruction, not on quality. It is well possible that exceptionally high quality mandatory instruction has a positive impact on learning that exceeds the negative impact of its reducing TSS. We also did not differentiate between different teaching formats, although it is likely that their impact on learning outcome is different. Schmidt e al. report that the strong inverted relation between lecture time and graduation rate was found to be weaker for other scheduled activities like practical training and tutorials [6]. In summary, when reducing quantity of instruction, it is important to carefully choose which formats to keep as guidance and stimulants for TSS and which formats to cut down on because they rather impede effective TSS.

## Conclusions

In conclusion, we found that the TSS/QI ratio as a measure of effectiveness of instruction is too low during the internal medicine and the surgical semesters. Furthermore, our data suggest that the time students spend on self study can be positively influenced by higher test frequency. We therefore propose a reduction of compulsory lessons to provide more time for self-study and an increase of test frequency as an incentive to make use of this time. These two key points have to be taken into account when implementing future curricular changes.

## Competing interests

The authors declare that they have no competing interests.

## Authors' contributions

SM, NW, VS, AH, GP and CK contributed to the design of survey 3, conduction of survey 3, statistical analysis of survey 3, interpretation of data of surveys 1-3 and have been involved in drafting the manuscript. MP contributed to data acquisition and statistical analysis of surveys 1 and 2. MH contributed to data acquisition, statistical analysis and data interpretation of surveys 1 and 2 and was involved in revising the manuscript. MS contributed to the design of surveys 1-3, statistical analysis of survey 1-2, interpretation of data of surveys 1-3 and has been involved in revising the manuscript. RS: contributed to the design of surveys 1-3, statistical analysis of survey 1-2, interpretation of data of surveys 1-3 and has been involved in revising the manuscript. All authors read and approved the final version of the manuscript.

## Authors' information

SM graduated from LMU medical school and recently started her residency at the Institute of Clinical Radiology of the University of Munich. She is involved in undergraduate medical education in Radiology.

NW graduated from LMU medical school and recently started her residency in the Department of Pediatric Oncology/Hematology, Charité Universitätsmedizin, Berlin.

VS graduated from LMU medical school. He is a research fellow in the Department of Surgery at Brigham and Women's Hospital/Harvard Medical School. His research focuses on trauma immunology.

AH graduated from LMU medical school. She recently started her residency in Internal Medicine at the University of Munich.

GP graduated from LMU medical school and recently started residency in the Department of Anesthesiology, Charité Universitätsmedizin, Berlin.

CK is a LMU Medical School graduate. He recently started his Plastic & Reconstructive Surgery residency in the Department of Surgery at the LMU Medical Center Grosshadern. He is involved in curricular planning and the conduction of an internal preparation course for the German licensing examination at our University.

MH studied information technology in Munich and has worked in the workgroup for medical education at LMU Munich for 15 years. He has started the development of a case-based learning system and is currently responsible for quality management and faculty development concerning medical assessments.

MP studied pedagogy, psychology and history in Munich. She is a research assistant in the workgroup for medical education at LMU Munich where she is responsible for the evaluation of the clinical curriculum.

MS is a general surgeon with an interest in inflammatory bowel disease and a medical educator with an interest in teaching with simulations. The current paper was stimulated by the notion that undergraduate students might be impaired by an overdose of contact time.

RS is Attending Physician and Educator. He currently finishes the Master of Medical Education (MME) program at the University Heidelberg. He is responsible for the Curriculum in Internal Medicine at the University Munich.

SM, NW, VS, AH, GP and CK were working together on this project of curriculum evaluation with regard to future reforms during their final year of medical school. They were supervised by MS and RS, who are senior faculty members in charge of the surgical and medical undergraduate medical education at our medical school, respectively.

## Pre-publication history

The pre-publication history for this paper can be accessed here:

http://www.biomedcentral.com/1472-6920/11/62/prepub
